# Ostéotomie de Gauthier et fixation par ostéo-sutures dans le traitement de la maladie de Freiberg

**DOI:** 10.11604/pamj.2018.29.33.8383

**Published:** 2018-01-16

**Authors:** Ahmed Daoudi, Najib Abbassi, Mounir yahyaoui, Omar Agoumi, Abdeljaouad Najib, Abdelkarim Daoudi, Hicham Yacoubi

**Affiliations:** 1 Service de Traumato-orthopédie du CHU Mohammed VI, Oujda, Maroc

**Keywords:** Freiberg, 2ndmetatarsal head, Gauthier’s osteotomy, bone suture, Freiberg, 2ème tête métatarsienne, ostéotomie de Gauthier, ostéosuture

## Abstract

La maladie de Freiberg est une ostéochondrose des têtes métatarsiennes, touchant le plus fréquemment la deuxième tête. Les auteurs rapportent à travers une étude rétrospective étalée sur trois ans, une série de 06 patientes, dont l’âge moyen est de 19,8 ans, opérées par une ostéotomie de soustraction dorsale selon Gauthier avec une fixation par des ostéosutures. Les résultats de cette technique sont satisfaisants vue que la majorité de nos patientes ont retrouvé l’indolence et ont récupéré une activité quotidienne habituelle. Les auteurs ont conclu que la fixation par des ostéosutures est une méthode fiable et peu coûteuse.

## Abstract (en)

Freiberg’s disease is an osteochondrosis of the metatarsal heads mainly affecting the second metatarsal head. This study was conducted over a 3 year period. It reports 6 cases of patients, whose average age was 19.8 years, who had undergone Gauthier’s dorsal subtraction osteotomy with fixation using osteosutures. The outcome of this technique was satisfactory. Indeed, the majority of patients had no pain and recovered normal daily activity. This study highlights that fixation using osteosutures is a reliable and inexpensive method.

## Introduction

La maladie de Freiberg-Kohler ou l’ostéochondrose des têtes métatarsiennes. Quoique rare, elle occupe le 4^ème^rang après l’atteinte de la tête fémorale, du condyle fémoral et celle de la tête humérale [1]. La prise en charge chirurgicale est dite polymorphe. Le nombre limité de séries dans la littérature n’a pas encor permis d’aboutir à un consensus précis. Communément, la chirurgie n’est indiquée qu’après l’échec d’un traitement conservateur bien conduit, en passant toujours par les mesures hygiéno-diététiques, le traitement médical à base des antalgiques et des anti-inflammatoires non stéroïdiens, les infiltrations et les orthèses plantaires. Les auteurs rapportent une série de 06 cas d’ostéonécrose de la 2^ème^ tête métatarsienne traité par une ostéotomie dorsale de soustraction selon Gauthier, avec une fixation par ostéo-sutures au fils résorbable.

## Méthodes

Sur une période de 03 ans, allant de Janvier 2012 au mois Décembre 2014, nous avons opéré 06 cas de maladie de Freiberg de la 2^ème^ tête métatarsienne. Il s’agit de 06 patientes dont l’âge moyen est de 19,8 ans (17-28 ans). Le tableau clinique commun était une métatarsalgie statique du 2^ème^ rayon, une gêne fonctionnelle et une limitation des activités quotidiennes habituelles. Deux de nos malades accusaient la notion d’un traumatisme ancien de l’avant pied dans l’apparition des symptômes. Le traitement chirurgical a était indiqué lors de la persistance des douleurs après l’échec d’un traitement conservateur bien mené pendant au moins 03 mois. Le stade radiologique avancé établis selon la classification de Smilie [2] nous a encore motivés d’opérer. Deux cas avaient un stade V et quatre cas avaient un stade IV. Toutes nos patientes ont été évaluées cliniquement et radiologiquement en pré et en post chirurgie par le score LMPI selon Kitaoka et al [3]. Le recul actuel de notre série est de 06 à 40 mois avec une moyenne de 20,2 mois.

### Technique chirurgicale: Ostéotomie de Gauthier

**Figure 1:**
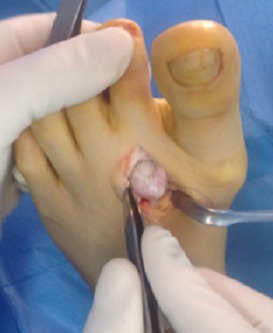
Aspect per-opératoire de l’hypertrophie synoviale et la destruction du cartilage articulaire

L’intervention se déroulait sous anesthésie locorégionale, le patient est installé en décubitus dorsal, l’utilisation de garrot pneumatique était systématique. La voie d’abord dorsale de 2-3cm, longitudinale et situé à cheval de l’articulation métatarsophalangienne (MTP), les tendons extenseurs sont écartés en externe et la capsule articulaire est incisée dans le sens de la voie d’abord. Le premier temps consiste à un débridement articulaire, résection des fragments ostéo-chondraux libres ainsi des zones hyperplasiques de la synoviale et un lavage articulaire (Figure 1).

**Figure 2:**
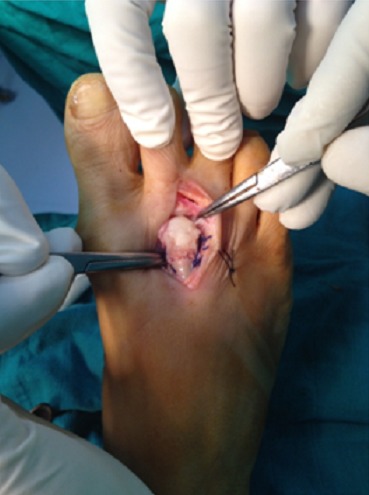
La fixation par un cadrage en U au fils résorbable

Le deuxième temps est une ostéotomie dorsale de soustraction d’un coin à base dorsale réalisé au niveau de la métaphyse métatarsienne pratiquement à ras du cartilage. La coupe conservait systématiquement une charnière plantaire. Un mouvement de rotation proximale mettait le cartilage plantaire sain de la tête en regard de la base de la première phalange. La fixation par un cadrage en U à l’aide du fil à résorption lente (Figure 2), puis la capsule articulaire suturée suivie d’une fermeture cutanée.

**Suites postopératoires**: Une immobilisation par une attèle postérieure avec une mise en décharge totale a était préconisé pendant 06 semaines. La rééducation et l’appui partiel sont ensuite débutés, alors que l’appui total n’a était autorisé qu’après la consolidation obtenu entre la 10^ème^ et la 12^ème^ semaine.

## Résultats

**Figure 3:**
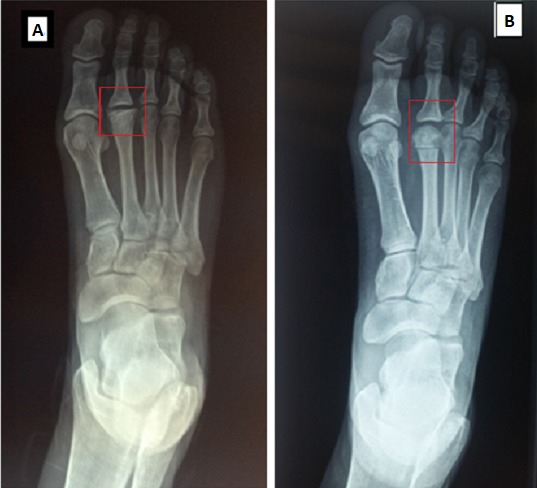
Radiographie standard; (A) Freiberg de la 2ème tête métatarsienne; (B) congruence articulaire après ostéotomie de Gauthier

La radiographie standard de contrôle, nous a permis d’objectiver la récupération de la sphéricité de la tête métatarsienne et de la congruence entre les deux surfaces articulaires (Figure 3). Le raccourcissement millimétrique de M2 situé entre 2 et 3mm, n’avait aucun retentissement sur la palette métatarsienne qui ce manifeste par des métatarsalgies de transfert. Le score LMPI en préopératoire se situait entre 51-70 avec une moyenne de 58,6. Alors que l’évaluation à notre recul actuel objective une amélioration significative du score que se situe entre 64 et 89 avec une moyenne de 78,5 (Tableau 1). La perte du secteur de mobilité de la MTP était constatée chez les 06 patientes, elle intéressait surtout la flexion palmaire avec une baisse de 15°(0-30°). Une seule patiente rapporte la persistance des douleurs, alors que toutes nos patientes ont récupéré leurs activités habituelles. Nous rapportons aucune complication liée à cette technique, notamment le déplacement secondaire par défaut mécanique, la pseudarthrose, la récidive de la nécrose ainsi que l’infection.

**Tableau 1:** Résultats de la série

Patiente	Age	Histoire du traumatisme	Stade	Suivie en mois	LMPI pré- op	LMPI post- op
1	18	non	IV	06	64	82
2	19	non	V	06	60	70
3	17	oui	IV	18	51	82
4	18	non	IV	28	53	84
5	19	non	IV	23	70	89
6	28	oui	V	40	54	64
Moyenne	19.8			20,2 mois	58,6	78,5

## Discussion

Initialement décrite en 1914, Freiberg [4] rapporte une série de six patients présentant une infraction de la deuxième tête métatarsienne. Depuis, de nombreuses publications s’intéressaient à l’explication physiopathologique de l’affection. La théorie traumatique et vasculaire est retenue, mais plusieurs auteurs sont convaincus du caractère étiopathogénique multifactoriel [5]. C’est une pathologie de la jeune femme, avec un sexe ratio de 5F/1H. Le pic de fréquence se situe entre 11 et 17 ans. La localisation sur le deuxième métatarse est de 68%, 27% au niveau du 3^ème^, 3% au niveau du 4^ème^ et de façon encore plus rare au niveau de la 5ème tête métatarsienne [6]. L’atteinte du 1^er^ rayon est exceptionnelle [7].

L’ostéotomie dorsale de fermeture, décrite initialement par Gauthier et ElBaz en 1979 [8], nous semble logique, simple et conservatrice concernant les surfaces articulaires. Les résultats sont satisfaisants vis-à-vis des douleurs avec une petite limitation de la mobilité concernant plus la flexion qui n’altère en aucun cas la fonction articulaire. Cette conclusion concorde avec la série de MB Al-Ashhab et al, 2013 [9] et de celle de Katcherian, 1994 [10]. En plus, la fixation par du fils résorbable nous a permis d’obtenir une stabilité satisfaisante à la consolidation, d’éviter un deuxième temps chirurgical d’ablation d’autant plus que c’est une chirurgie à faible cout. On a constaté une amélioration du score LMPI lorsque le diagnostic est établi à un âge jeune, ainsi que l’intervention est immédiatement réalisée après l’échec du traitement conservateur. Tant qu’on opère à un âge jeune, tant qu’on obtient un bon remodelage articulaire et une récupération rapide des activités ultérieurs. Tant que le stade radiologique est avancé, tant que les dégâts articulaires sont plus importantes et le résultat est moins bon. Dans notre série, la seule patiente insatisfaite du résultat avait 28 ans et un stade V de l’infraction.

En comparaison à d’autres séries. MB Al-Ashhab et al [9] Rapportent des bons résultats fonctionnels après l’ostéotomie de Gauthier et fixation par des broches de Kirchner dans leur série de 10 patientes. Gauthier et ElBaz [8] dans leur série de 53 patients opérés par la même technique, objectivent la persistance des douleurs chez un seul patient. Lee et al [11] rapportent une série de 12 patients traités par ostéotomie de gauthier avec une fixation par des broches résorbables. Un seul patient insatisfait avait présenté un retard de consolidation avec un gonflement persistant. Capar et al [12] dans une série de 19 patients. Deux patients gardaient des douleurs dont un avait un stade V et le deuxième avait un stade IV. Dans toutes les séries, la consolidation a était toujours obtenu, même si elle est retardée. La douleur est soulagée et la récupération des activités quotidiennes habituelles a était constatée chez la majorité des patients.

## Conclusion

L’ostéotomie de Gauthier avec une fixation par des ostéo-sutures associée à un débridement et une synovectomie est une technique simple permettant la restauration de la congruence articulaire de la MTP. On conclu comparativement à la littérature que l’âge jeune et le stade radiologique précoce restent les seuls garant des bons résultats fonctionnels.

### Etat des connaissances actuelle sur le sujet

- L’ostéotomie de soustraction dorsale selon Gauthier avec une fixation par du matériel d’ostéosynthèse (Broches de kirschner, mini-vis, mini-plaque);
- Traitement chirurgical: résection de la tête arthroplastie d’interposition, arthrodèse, lavage débridement articulaire.

### Contribution de notre étude à la connaissance

- Ostéotomie et fixation par du fils résorbable (Vicryl 2) ce qui permet d’éviter un 2^ème^ temps opératoire d’ablation du matériel;
- Technique peu couteuse;
- Méthode fiable permettant de débuter la rééducation sans aucun cas de lâchage des suture ou d’autres complications.

## Conflits d’intérêts

Les auteurs ne déclarent aucun conflit d'intérêt.

## Contributions des auteurs

Les patients ont été opéré par deux chirurgiens: Hicham Yacoubi et Omar Agoumi; Les patientes ont été vues en consultation par deux chirurgiens: Ahmed Daoudi et Mounir Yahyaoui; L’article est élaboré par: Ahmed Daoudi; L’article est corrigé par: Abdelkarim Daoudi, Abdeljaouad Najib, Najib Abbassi.
